# Biofilm dispersion by *Pseudomonas aeruginosa* requires relocation of BdlA to drive a switch in motility by dispersed cells

**DOI:** 10.1128/jb.00022-26

**Published:** 2026-08-12

**Authors:** Soyoung Park, Karin Sauer

**Affiliations:** 1 Department of Biological Sciences, Binghamton University 171431, Binghamton, New York, USA; 2 Binghamton Biofilm Research Center, Binghamton University 14787, Binghamton, New York, USA; National Institutes of Health, Bethesda, Maryland, USA

**Keywords:** biofilm dispersion, motility, *Pseudomonas aeruginosa*, BdlA, DipA, polar protein localization

## Abstract

Biofilm dispersion is a regulated process that enables bacteria to escape as free-living cells. This response coincides with matrix degradation, reduced cyclic di-GMP levels, heightened antibiotic susceptibility, and restored flagellar motility. While dispersed cells have been reported to be motile, the mechanisms enabling motility upon induction of dispersion remain unclear. Here, we explored the regulatory mechanism of how *Pseudomonas aeruginosa* biofilm cells switch their motility phenotype to disperse from biofilms. Our findings reveal that changes in motility gene expression are initiated at the periphery of biofilms during induced dispersion, in which increased expression of the flagellar gene *fliC* is controlled by the phosphodiesterase (PDE) DipA and the chemosensory protein BdlA. In response to the dispersion signal, BdlA forms dynamic clusters and relocates to the flagellated cell pole, where it interacts with and stimulates the PDE activity of DipA, ultimately leading to elevated *fliC* expression. These findings establish a mechanistic link between signal sensing and motility reversion, demonstrating that the spatiotemporal coordination of BdlA and DipA governs the motility switch during *P. aeruginosa* biofilm dispersion.

**IMPORTANCE:**

Our findings reveal a new regulatory mechanism in which dispersion signals drive BdlA relocation, activate DipA’s phosphodiesterase activity, lower cyclic di-GMP, and elevate *fliC* expression. Moreover, by showing that BdlA foci formation and co-localization with DipA occur in response to dispersion signals, we demonstrate that the spatiotemporal regulation of BdlA and DipA extends the Touch-Seed-and-Go model beyond surface attachment to encompass dispersion and, thus, the reversion from a sessile to a motile growth state.

## INTRODUCTION

Biofilms are surface-associated bacterial communities encased in a self-produced polymeric matrix composed of DNA, proteins, and exopolysaccharides (1–3). Biofilms are prevalent in nature and during infections, with the biofilm mode of growth offering several advantages, including being close to nutrients, facilitating the exchange of genetic material, and providing protection against various chemical and environmental stresses such as antimicrobial agents and shear forces (4). However, as the biofilm develops, its community becomes stratified, leading to decreased nutrient and oxygen availability in the interior and increased exposure to waste products and toxins. To cope with these challenges, bacteria employ regulatory mechanisms to escape from mature biofilms and transition to a free-living lifestyle, a process known as biofilm dispersion (5–7).

Biofilm dispersion by *Pseudomonas aeruginosa*, an opportunistic human pathogen, is an active process that is induced by various factors, including nutrient availability, environmental stressors and signals, and the accumulation of self-synthesized signaling molecules such as *cis*-2-decenoic acid (*cis*-DA) (6–11). Recent findings suggest that while the mechanisms for sensing dispersion signals are distinct, the downstream processes that lead to dispersion by *P. aeruginosa* biofilms depend on a shared pathway (12). The dispersion signals and cues are perceived by membrane-associated receptors such as DspS (for *cis*-DA) (12), NicD (for nutrients and amino acids) (13), and NbdA (for nitric oxide) (14), which, in turn, result in a series of post-translational modifications that ultimately result in an overall reduction in the levels of the intracellular signaling molecule cyclic di-GMP (c-di-GMP) (Fig. S1A). Specifically, the perception of signals induces activation of the chemotaxis transducer protein BdlA, which requires phosphorylation followed by non-processive proteolytic cleavage by the protease ClpD (13, 15, 16). Once activated, BdlA interacts with and stimulates the phosphodiesterase (PDE) activity of DipA (also referred to as Pch) and RbdA, thereby reducing intracellular c-di-GMP levels (16–18) (Fig. S1A). Inactivation of *dipA* and *rbdA* has previously been shown to impair dispersion by *P. aeruginosa* biofilms in response to various dispersion signals, including nutrients, nitric oxide, and *cis*-DA (12, 13, 17).

Induction of dispersion, combined with the reduction in c-di-GMP levels, has been linked to the unique transcriptome and traits of dispersed cells compared to biofilm cells in *P. aeruginosa* (17, 19–21). These include decreased expression of genes encoding the biofilm phenotype, such as antibiotic resistance and exopolysaccharide production, as well as upregulation of genes involved in matrix-degrading enzymes, metabolic and energy-generating pathways, and flagellar motility (22–24). However, the regulatory mechanisms that enable dispersed cells to adopt these traits to actively leave the biofilm are not fully understood.

The reduction in c-di-GMP levels upon induction of dispersion by the PDEs RbdA and DipA (16–18) has likewise been linked to a reversion in the mode of growth, supported by c-di-GMP levels inversely regulating biofilm formation and the motile, planktonic state (21, 25). In turn, dispersion by *P. aeruginosa* is assumed to coincide with a reversion from a non-motile to a motile phenotype, supported by observations of bacteria actively leaving the center of the biofilm structure during native dispersion (26). However, little is known about the role of flagella and swimming motility in biofilm dispersal, as reports on the requirement of flagella and swimming motility vary from being crucial, not always required, to dispersal occurring in the absence of significant motility. For example, Thormann and Spormann reported swimming motility not to be required for biofilm dispersion by *Shewanella oneidensis* MR-1 induced by arresting the medium flow in the biofilm system (27). In contrast, flagella-mediated swimming motility has been shown to be important for the dispersal of *Vibrio cholerae* biofilms (28) and mucin-induced *P. aeruginosa* biofilm dispersal (29).

In this study, we unravel the regulatory mechanism governing the reversion to a motile lifestyle during dispersion by dissecting the early regulatory events in biofilm dispersion that lead to the expression of the flagellar gene *fliC*, allowing dispersed cells to regain flagella-driven motility. Our findings indicated that changes in motility gene expression are crucial for the dispersion event and are initiated at the periphery of mature biofilms, where DipA and BdlA control the upregulation of *fliC*. We demonstrated that relocation of BdlA to DipA at the flagellated cell pole in response to dispersion signals resulted in a reduction of c-di-GMP levels, thereby enhancing *fliC* expression. Therefore, our findings reveal how dispersed cells adopt a motility phenotype and egress from mature biofilms.

## RESULTS

### Biofilm dispersion is associated with flagellar-dependent motility

**Fig 1:**
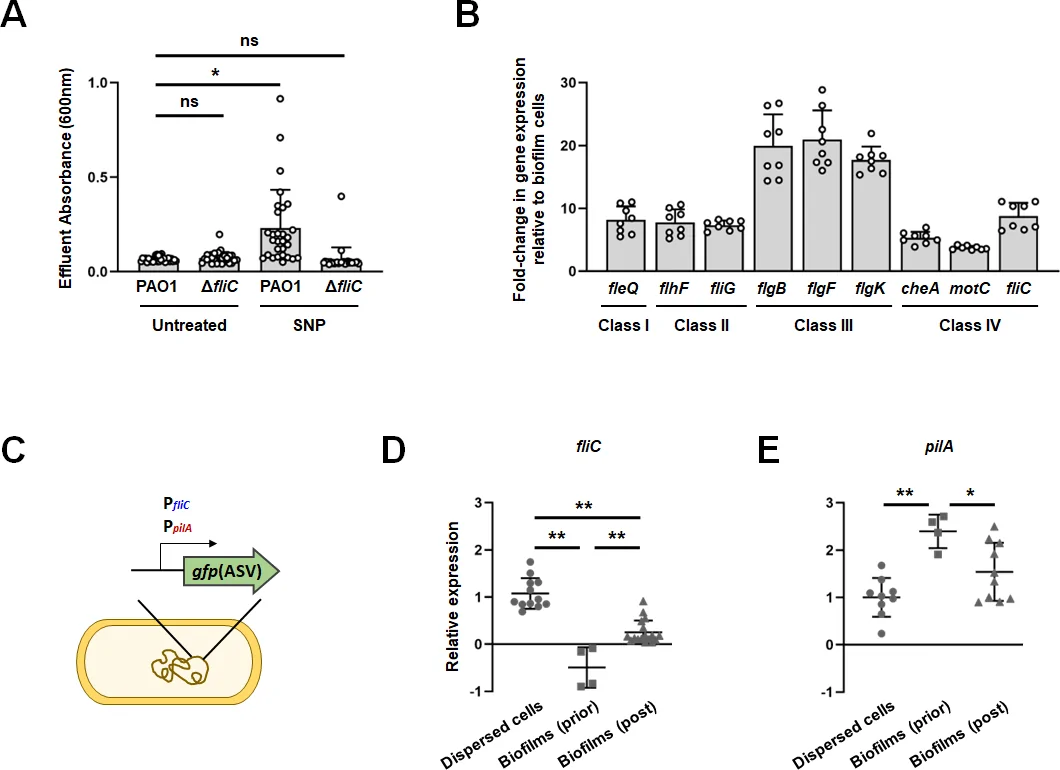
Motility gene expression during biofilm dispersion. Biofilms by *P. aeruginosa* PAO1 wild-type and *fliC* deletion strain were grown for 5 days in fivefold-diluted VBMM in tube reactors under flowing conditions. (**A**) Dispersion was induced upon exposure to 0.5 mM sodium nitroprusside (SNP) as a source of nitric oxide. Effluents from the tube reactors were collected for 30 min in 1-min intervals, and the absorbance was determined at 600 nm by spectrophotometry. Untreated biofilms were used as controls. The absorbance of effluents collected 15 to 25 min post-exposure of SNP or medium alone (untreated) was selected and quantified. (**B**) Transcript abundance of flagellar genes. RNA was obtained from biofilms and SNP-induced dispersed cells. The transcript abundances of flagellar genes were analyzed by qRT-PCR. Each column represents the transcript abundance of the genes in dispersed cells relative to biofilms. (**C–E**) Schematic representation of flagella (*fliC*) and pili (*pilA*) transcriptional fusion reporters (**C**). Relative *fliC* (**D**) and *pilA* (**E**) expression levels were determined using the reporters. Tube reactor-grown biofilms formed by each reporter strain, prior to or post-exposure to SNP, and SNP-induced dispersed cells were collected, followed by measurement of GFP and absorbance at 600 nm. GFP fluorescence was normalized to absorbance. All experiments were performed using biological triplicates with two technical replicates. Error bars indicate standard deviation. Statistical significance was assessed using one-way analysis of variance, followed by Tukey’s multiple comparison test. **, *P* < 0.001; *, *P* < 0.05; ns, not significant.

Based on the general assumption that non-motile bacteria in biofilms become motile upon the initiation of dispersion, we asked if flagellar motility is required for the dispersion response. We therefore assessed dispersion in a *fliC* mutant lacking flagella-driven motility. *fliC* encodes flagellin type B, essential for swimming and swarming (30). Biofilms by *P. aeruginosa* wild-type and the *fliC* mutant strain were grown for 5 days, followed by induction of dispersion in response to nitric oxide. Biofilm effluents were collected, and then the absorbance values of the effluents were determined at 600 nm. The quantitative analysis of the dispersion response indicated that exposure of wild-type biofilms to nitric oxide resulted in a significant increase in the effluent absorbance, whereas biofilms formed by the *fliC* mutant did not (Fig. 1A). The findings indicated that flagella-driven motility plays a crucial role in the dispersion response to nitric oxide.

### Biofilm dispersion is associated with increased flagellar gene expression

In *Pseudomonas*, over 40 genes are associated with flagella and chemotaxis, which are expressed and organized into four hierarchical levels (Classes I, II, III, and IV) to enable a highly ordered flagellar assembly process (30, 31). The *fliC* gene belongs to Class IV, which is expressed last. The finding of flagellar motility being required for dispersion prompted us to ask whether induction of dispersion coincides with bacteria becoming motile, likely through elevated expression and thus synthesis of the flagellum and/or the entire flagellar apparatus. We therefore assessed the expression of representative genes of each class by quantitative real-time PCR (qRT-PCR). The transcript abundance of Classes I–IV genes was significantly elevated in dispersed cells obtained in response to the dispersion signal nitric oxide, compared to *P. aeruginosa* PAO1 biofilm cells grown for 5 days in a tube reactor (Fig. 1B). Notably, flagellar genes belonging to Class I (*fleQ*), Class II (*flhF* and *fliG*), and Class IV (*cheA*, *motC*, and *fliC*) were increased by up to sevenfold in dispersed cells, while genes in Class III (*flgB*, *flgF*, and *flgK*) showed an average increase of ~19-fold (Fig. 1B). Our results indicated that all classes of genes associated with flagella and chemotaxis are upregulated during dispersion, suggesting a requirement for *de novo* synthesis of flagella to enable a switch in motility post-induction of dispersion.

### Spatiotemporal expression patterns of flagella and pili within biofilms upon induction of dispersion

The finding of flagellar genes showing transient induction during dispersion prompted us to examine the timing of their activation following exposure to dispersal cues. We used a *fliC* promoter fused to the unstable GFP(ASV) reporter (Fig. 1C) and quantified fluorescence in 5-day-old *P. aeruginosa* wild-type biofilms before and after nitric-oxide–induced dispersion. A *pilA* transcriptional fusion served as a control, as Type IV pili promote surface attachment and biofilm formation (29). *fliC* reporter activity increased significantly in dispersed cells compared with intact and post-dispersion biofilms (Fig. 1D). In contrast, *pilA* reporter activity decreased in dispersed and post-dispersion biofilms relative to intact biofilms (Fig. 1E).

**Fig 2:**
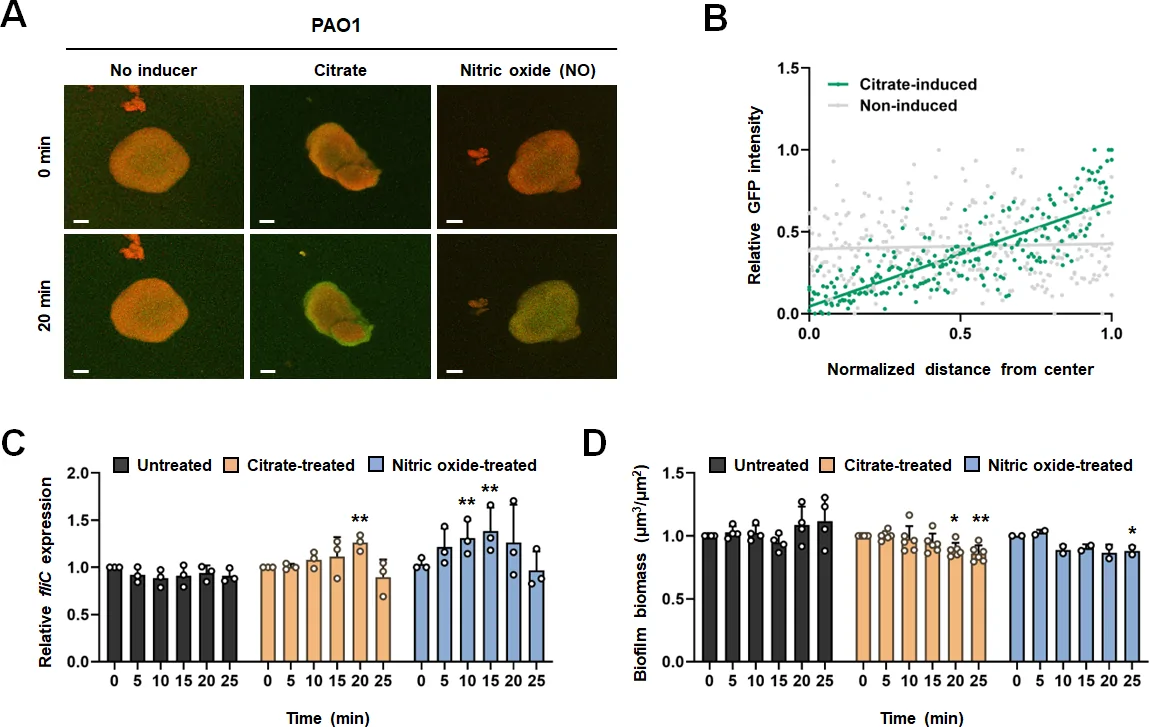
Expression patterns of *fliC* by wild-type biofilms upon induction of dispersion. Biofilms formed by wild-type PAO1 *fliC* reporter strains were grown for 4 days in fivefold-diluted VBMM using flow cells under constant flow conditions and stained with SYTO 62 prior to microscopy for biomass. Subsequently, dispersion was induced by adding 20 mM sodium citrate or 0.5 mM sodium nitroprusside (SNP), and the biofilms were monitored at 5-min intervals to assess changes in the *fliC* expression and in biomass (see also Fig. S1). (**A**) Representative confocal images at 0 and 20 min are shown. Bars, 25 μm. (**B**) Spatial distribution of *fliC* expression within microcolonies formed by PAO1 was determined. Line scan analysis shows the relative GFP intensity from the center (0) to the edge (1) of the microcolonies. Regression lines (solid lines) were fitted to the pooled data points from three independent microcolonies (*n* = 3) to compare *fliC* expression levels 20 min post-citrate exposure (green) and non-induced controls (light gray). Raw GFP intensity (*fliC* expression) was normalized by subtracting the minimum intensity and scaling each value to the full intensity range (maximum minus minimum), yielding a relative fluorescence measure at each point. (**C and D**) Time-dependent changes were determined by COMSTAT analysis. (**C**) Changes in *fliC* expression (GFP intensity divided by SYTO 62) in wild-type biofilms left untreated or exposed to dispersion signal nitric oxide and citrate. (**D**) Total biomass of wild-type biofilms left untreated or exposed to dispersion signal nitric oxide and citrate. All experiments were performed in triplicate. For quantitative image analysis, at least eight images per replicate were examined. Statistical significance relative to time = 0 was assessed using an unpaired, two-tailed *t*-test. *, *P* < 0.05; **, *P* < 0.01.

To determine the spatial and temporal dynamics of *fliC* and *pilA* expression upon induction of dispersion, we next visualized the reporter activity in biofilms prior to and post-exposure to two dispersion signals, nitric oxide and citrate. *fliC* expression appeared to be low and somewhat diffused in intact biofilms as well as biofilms exposed to the dispersion signal citrate for 10 min (Fig. 2A; Fig. S1B). By ~15–20 min post-citrate exposure, GFP fluorescence increased at the microcolony periphery, followed by a decline by 25 min (Fig. 2A; Fig. S1B). Line-scan analysis further showed that *fliC* induction localized to the microcolony edge, whereas untreated biofilms exhibited no temporal change (Fig. 2B). Notably, no significant changes in *fliC* expression were observed in non-induced microcolonies over the same period, as demonstrated by confocal images, COMSTAT quantification, and line-scan analysis (Fig. 2A through C). Quantitative analysis using COMSTAT confirmed a significant increase in *fliC* expression within ~20 min post-induction of dispersion, followed by a subsequent decrease (Fig. 2C; Fig. S1C). This decrease in *fliC* gene expression coincided with a significant loss of biofilm biomass, indicating dispersion events (Fig. 2D; Fig. S1D). Image analysis indicated loss of biofilm biomass to occur at the biofilm edge (Fig. S1C). In contrast, *pilA* expression was detectable primarily at the periphery of untreated biofilms (Fig. S1B). Moreover, induction of dispersion coincided with an overall and continued reduction of *pilA* expression, with quantitative COMSTAT analysis confirming a significant reduction of *pilA* expression ~20 min post-induction of dispersion (Fig. S1).

Exposure of wild-type biofilms to nitric oxide induced a comparable *fliC* expression pattern (Fig. 2A), indicating a conserved transcriptional response to distinct dispersion signals. COMSTAT analysis confirmed a significant transient increase in *fliC* expression post at ~20 min, coinciding with a marked loss of biomass consistent with active dispersion (Fig. 2B and C).

Together, these data show that dispersion cues elicit a rapid, peripheral burst of flagellar gene expression accompanied by a concurrent decrease in pili gene expression. The subsequent decline in both reporters reflects biomass loss as cells disperse. These findings support a model in which dispersion, triggered by an exogenous dispersion signal, initiates at the biofilm edge, marked by elevated *fliC* expression and peripheral biomass loss (13, 16).

A distinct feature of native biofilm dispersion is the formation of central voids, indicating dispersion to be initiated in the interior of biofilms, rather than the periphery (5, 11, 12). It is noteworthy that intensive *fliC* expression was observed at the central voids within the microcolony (Fig. S2), further supporting that flagellar gene expression is prominently increased in cells located at the site where dispersion occurs.

### DipA plays a role in flagellar gene expression during biofilm dispersion

**Fig 3:**
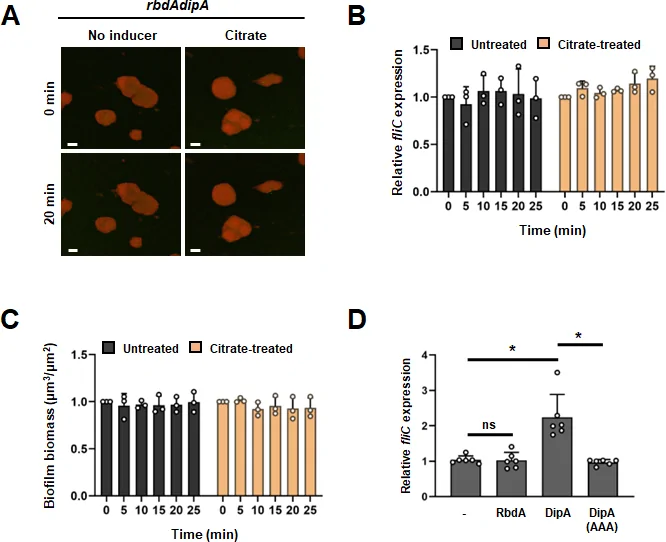
Expression patterns of *fliC* by *rbdAdipA* mutant biofilms upon induction of dispersion. Biofilms formed by the *rbdAdipA* double-mutant *fliC* reporter strain were grown for 4 days in fivefold-diluted VBMM using flow cells under constant flow conditions and stained with SYTO 62 prior to microscopy for biomass. Subsequently, dispersion was induced by adding 20 mM sodium citrate, and the biofilms were monitored at 5-min intervals to assess changes in the *fliC* expression and in biomass. (**A**) Representative confocal images at 0 and 20 min are shown. Bars, 25 μm. (**B and C**) Time-dependent changes were determined by COMSTAT analysis. At least eight images per replicate were used for quantitative image analysis. No significant difference was detected. (**B**) Changes in *fliC* expression (GFP intensity divided by SYTO 62) in *rbdAdipA* biofilms left untreated or exposed to the dispersion signal citrate. (**C**) Total biomass of *rbdAdipA* biofilms left untreated or exposed to the dispersion signal citrate. (**D**) Relative *fliC* expression levels were determined using biofilms formed by wild-type *fliC* reporter strains harboring an empty vector or vectors expressing *rbdA*, *dipA*, or an inactive *dipA*(AAA). All experiments were performed in triplicate. Statistical significance was assessed using one-way analysis of variance, followed by Tukey’s multiple comparison test. *, *P*  <  0.001; ns, not significant.

Flagellar gene expression is linked to c-di-GMP levels (21, 32), with FleQ positively regulating the flagellar genes at low c-di-GMP levels (31). As dispersion coincides with an overall reduction in c-di-GMP levels (7, 17, 21), we reasoned that a c-di-GMP modulation protein, specifically a phosphodiesterase (PDE), may contribute to the activation of flagellar gene expression. As two active PDEs, DipA and RbdA, have been reported to be required for dispersion (7, 17, 18), we asked whether *fliC* expression depends on DipA and/or RbdA. First, we examined *fliC* expression changes in biofilms formed by the Δ*rbdA*Δ*dipA* mutant strain in response to the dispersion signal citrate. As shown in Fig. 3A and B, no significant change in *fliC* expression was observed following exposure to citrate. Likewise, no loss of biofilm biomass in response to citrate was noted (Fig. 3C). The findings suggested that either one or both PDEs are involved in regulating *fliC* expression during the dispersion response.

To determine whether one or both PDEs contribute to *fliC* expression, we next examined the effects of overproducing DipA or RbdA. Biofilms were grown for 3 days under constant flow conditions and harvested, and relative *fliC* transcript levels were quantified. Overproduction of RbdA did not alter *fliC* expression compared with the empty-vector control (Fig. 3D). In contrast, DipA overproduction resulted in an approximately twofold increase in *fliC* expression (Fig. 3D). This increase was absent in biofilms expressing a catalytically inactive DipA variant carrying alanine substitutions in the EAL motif (DipA(AAA)), which cannot degrade c-di-GMP (17) (Fig. 3D). These findings suggested that DipA, but not RbdA, enhances *fliC* expression, consistent with DipA-mediated lowering of c-di-GMP to promote flagellar gene induction during dispersion.

### Polar localization of DipA is independent of the mode of growth and intracellular c-di-GMP levels

DipA (Pch) has recently been shown to be responsible for c-di-GMP heterogeneity after cell division by asymmetrically positioning to the flagellated pole, where it interacts with and is activated by CheA, a receptor-associated kinase of chemotaxis machinery located at the flagellated pole (33). In turn, polar localization of DipA was found to be associated with low cellular c-di-GMP after cell division (33), while non-flagellated daughter cells with high levels of c-di-GMP lacked polar DipA (33, 34).

**Fig 4:**
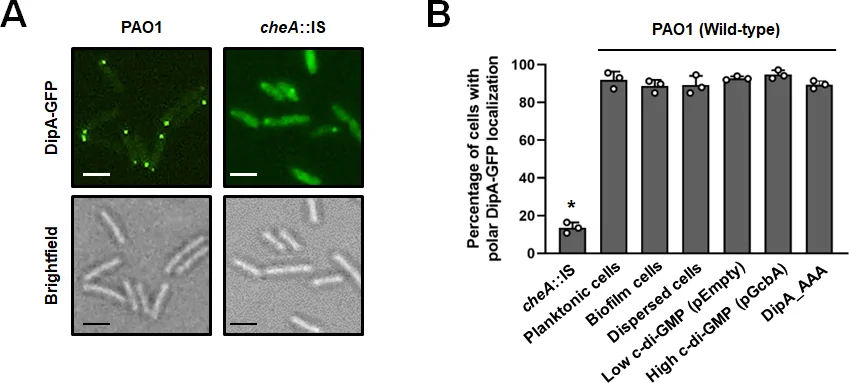
Polar localization of DipA is CheA-dependent. GFP-fused DipA (DipA-GFP) was expressed in the wild-type *P. aeruginosa* PAO1 and the *cheA* transposon mutant (*cheA*::IS). (**A**) DipA subcellular localization was observed using fluorescence microscopy. (**B**) The percentage of cells exhibiting polar localization of DipA-GFP in each strain and condition was quantified. At least three images per replicate representing a total of 1,000 cells were examined. Asterisks indicate statistically significant differences relative to planktonic wild-type cells (*, *P*  <  0.001 based on Student’s *t*-test).

Given the link between c-di-GMP, motility, and the polar localization of DipA, we reasoned that DipA might be diffuse under biofilm conditions but relocalize to the pole upon induction of dispersion (and low c-di-GMP levels). To track the subcellular location of DipA, we used a plasmid-encoded DipA-GFP fusion (DipA-GFP). Consistent with previous reports (33), DipA localized to the cell pole in planktonic cultures (Fig. 4A), with ~92% of cells exhibiting polar foci (Fig. 4A and B). Inactivation of *cheA* reduced polar DipA localization to <20% (Fig. 4B), confirming CheA-dependent targeting of DipA to the flagellated pole (33–35). Polar DipA foci were also observed in biofilm cells and in dispersed cells obtained in response to the dispersion signal nitric oxide (Fig. 4B; Fig. S3). However, no significant difference in DipA distribution was noted, with the DipA-GFP fusion protein being predominantly located at the cell pole in approximately 89% of dispersed cells and ~88% of the biofilm cells (Fig. 4B). The elevated frequency of polar DipA observed here likely reflects plasmid-borne *dipA-gfp* overexpression, whereas prior studies used chromosomally integrated *dipA* constructs that produce lower protein levels (33, 34). However, despite overproduction of DipA, localization of DipA remains specific and biologically relevant (CheA-dependent). Neither modulation of c-di-GMP levels, by overexpressing *gcbA* encoding an active diguanylate cyclase GcbA (36), nor overproducing inactive DipA (DipA_AAA) altered the polar localization of DipA (Fig. 4B).

### BdlA relocates and forms dynamic clusters in response to a dispersion signal

Our findings so far indicate that activation of *fliC* expression is independent of DipA’s spatial distribution. This raised the question of how DipA becomes activated during dispersion to generate low c-di-GMP levels and promote flagellar gene expression. Prior studies indicated that activated BdlA not only interacts with DipA but also stimulates the PDE activity of DipA upon induction of dispersion (7, 13, 16). This led us to hypothesize that BdlA may contribute to increased *fliC* expression by co-localizing with DipA upon induction of dispersion and thereby enhancing the PDE activity of DipA.

**Fig 5:**
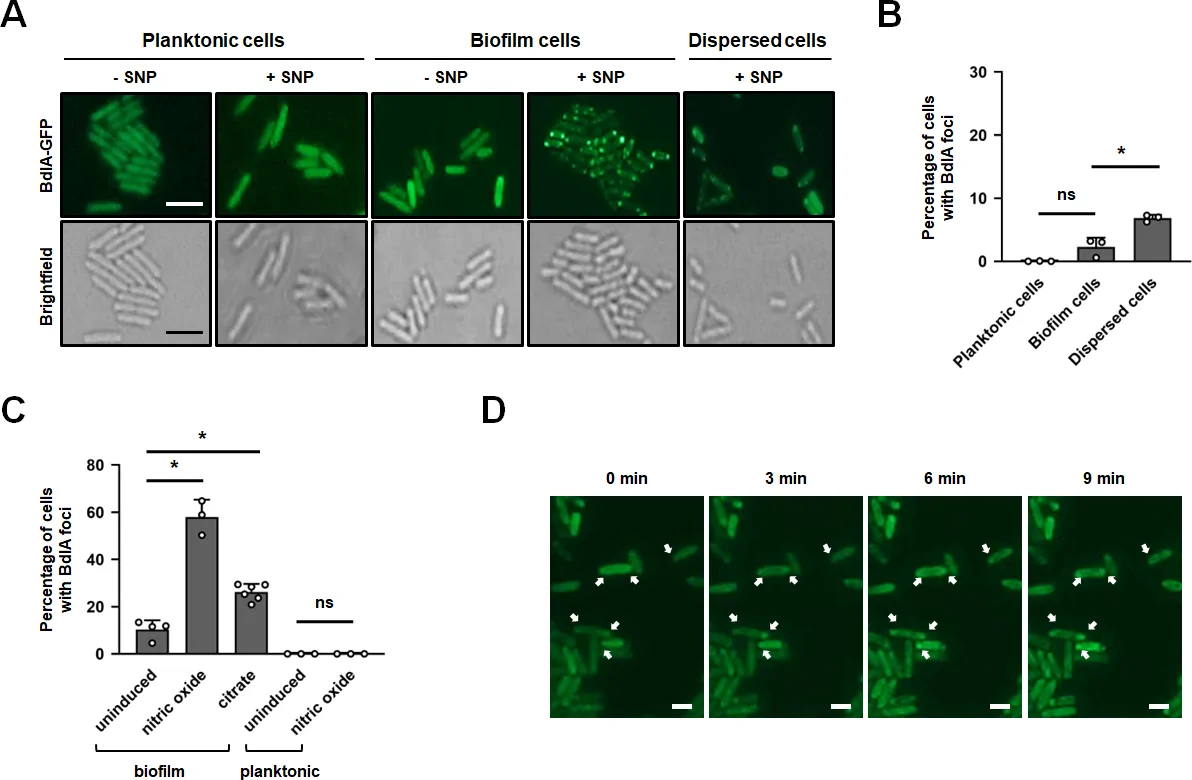
BdlA forms clusters and relocates to the cell pole during biofilm dispersion. GFP-fused BdlA (BdlA-GFP) was expressed in *P. aeruginosa* PAO1, and its localization was observed using fluorescence microscopy. Planktonic cells and tube reactor-grown biofilms were harvested. These cells were either exposed to nitric oxide (SNP) after harvesting or left untreated. Dispersed cells were obtained by collecting biofilm effluents after SNP exposure. (**A**) Representative images and (**B**) quantification of the number of cells showing fluorescence foci formed by BdlA-GFP are shown from three biological replicates. At least three images per replicate representing a total of 1,000 cells were analyzed. Bars, 2 μm. (**C**) Quantification of the number of foci formed by BdlA-GFP per wild-type biofilm cell in the absence and presence of nitric oxide over the course of 30 min. At least two images per replicate and a minimum of 100 cells per image were analyzed. (**D**) Time course of BdlA foci formation prior to and post-exposure to nitric oxide. White arrows indicate BdlA foci. Bars, 2 μm. Statistical significance was assessed using an unpaired, two-tailed *t*-test *, *P*  <  0.01; ns, not significant.

To assess BdlA localization, we expressed a C-terminal BdlA-GFP fusion in planktonic, biofilm, and nitric-oxide–induced dispersed cells (Fig. 5A and B). BdlA-GFP was diffusely distributed in both planktonic and biofilm cells. In contrast, dispersed cells exhibited distinct multi-spot BdlA foci in ~10% of the population (Fig. 5A and B).

To assess whether the dispersion signal triggers foci formation of BdlA in dispersed cells, we examined BdlA localization in biofilm cells exposed to nitric oxide or left untreated. Biofilm cells expressing *bdlA-gfp* were harvested, with half of the biofilm sample subjected to nitric oxide for 20 min and half serving as controls. The resulting samples were spotted onto agar pads for subsequent microscopic analysis. In control biofilms, BdlA-GFP remained diffusely distributed throughout the cells (Fig. 5A). Quantitative analysis of ~1,000 biofilm cells per experiment revealed up to 10% of untreated biofilm cells harboring BdlA foci (Fig. 5C). Exposure of biofilms to nitric oxide coincided with BdlA relocalization, with ~58% of cells forming distinct BdlA foci and ~39% showing polar accumulation (+ SNP in Fig. 5A and C). The higher proportion of BdlA foci in nitric-oxide–treated biofilm cells (~58%, Fig. 5C) compared with dispersed cells (~10%, Fig. 5B) likely reflects the more uniform and synchronous exposure of intact biofilms to the dispersion cue, enabling a stronger and more coordinated BdlA response. A similar but more modest increase in BdlA foci formation was noted when biofilms were exposed to the dispersion signal citrate (Fig. 5C).

To determine whether BdlA relocalization in response to the dispersion signal is growth mode–dependent, we examined BdlA localization in planktonic cells exposed to nitric oxide or left untreated. In untreated planktonic cells, BdlA-GFP remained diffusely distributed, and nitric oxide exposure did not alter this pattern (+ SNP in Fig. 5A and C). These results indicate that BdlA foci form only in response to the dispersion signal under biofilm growth conditions, demonstrating a growth mode–dependent relocalization.

We next determined the timing of BdlA relocalization following exposure to the dispersion signal nitric oxide. To do so, we monitored BdlA-GFP localization in biofilm cells at 3-min intervals over a 12-min period. BdlA foci appeared rapidly, becoming detectable within 3 min of exposure (Fig. 5D). Continued nitric oxide treatment led to additional foci formation over time (Fig. 5D; Fig. S6).

Previous studies have linked BdlA activation to dispersion-signal sensing (15, 16). As BdlA foci formed in response to the dispersion signal (Fig. 5), we next asked whether BdlA activation status influences foci formation. However, BdlA foci formed regardless of activation state, as a GFP-tagged BdlA_M130I variant, which is defective in processing and activation (15), still formed foci upon exposure to the dispersion signal (Fig. S4). These results indicate that BdlA repositioning is activation-independent but likely precedes and enables subsequent BdlA activation. While active BdlA was not required for relocalization, *fliC* expression required active BdlA. Biofilms overexpressing the constitutively active BdlA_G31A variant (16) showed increased *fliC* expression relative to vector controls (Fig. S5).

### BdlA co-localizes with DipA at the flagellated pole

**Fig 6:**
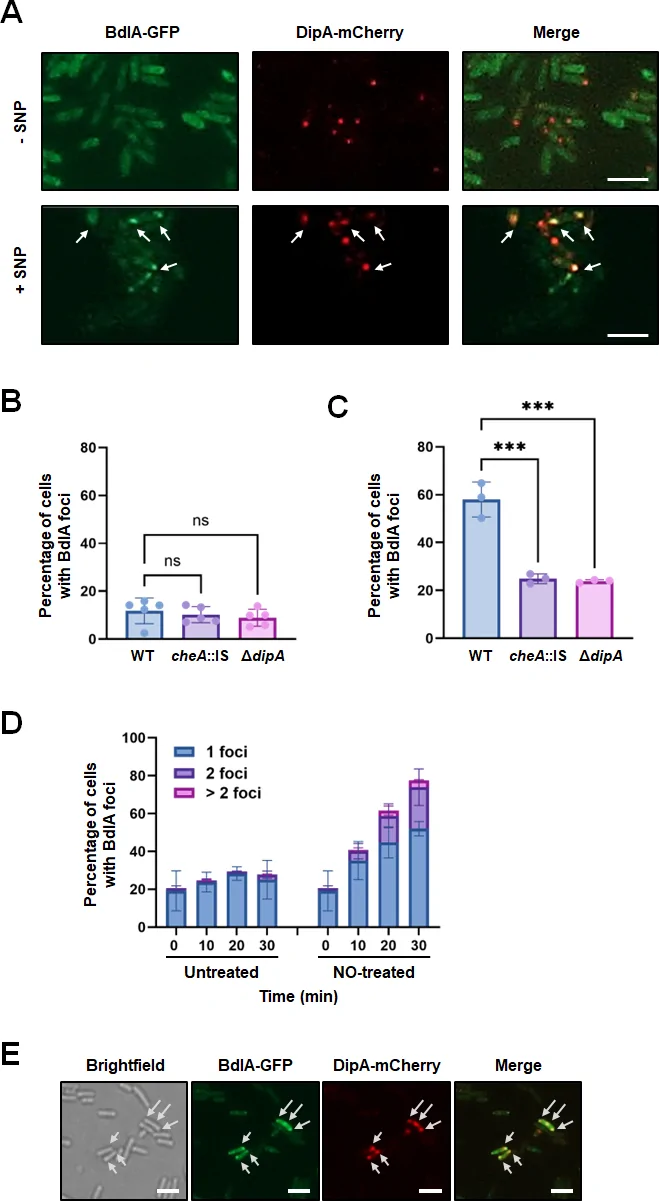
BdlA co-localizes with DipA. (**A**) Localization of BdlA-GFP and mCherry-fused DipA (DipA-mCherry) in biofilm cells under nitric oxide exposure or untreated conditions. Biofilms were exposed to nitric oxide (SNP) after harvesting. White arrows indicate co-localization parts. Bars, 5 μm. SNP was used as a source of nitric oxide. (**B and C**) Quantification of the number of cells showing fluorescence foci formed by BdlA-GFP in biofilms formed by wild type, Δ*dipA*, and Δ*cheA* in the (**B**) absence and presence (**C**) of dispersion signal nitric oxide. Statistical significance was assessed using one-way analysis of variance, followed by Dunnett’s multiple comparison test. ***, *P*  <  0.001; ns, not significant. (**D**) Quantification of the number of foci formed by BdlA-GFP formed per wild-type biofilm cell in the absence and presence of nitric oxide over the course of 30 min. (**B–D**) At least three images per replicate representing a total of 1,000 cells were analyzed. (**E**) Representative image showing co-localization of multiple BdlA-GFP and mCherry-fused DipA (DipA-mCherry) in biofilm cells post exposure to nitric oxide. White arrows indicate co-localization. Bars, 2 μm.

Our results so far demonstrate that BdlA relocates to the pole upon dispersion signaling to drive flagellar gene expression. To determine whether BdlA moves specifically to the flagellated pole, we next examined whether BdlA co-localizes with DipA, using a strain co-expressing *dipA* fused to mCherry and *bdlA* fused to GFP. In the absence of the dispersion signal nitric oxide, biofilm cells demonstrated polar foci of the DipA-mCherry fusion protein, while BdlA-GFP remained diffused (Fig. 6A). Following exposure to nitric oxide, however, BdlA and DipA were found to co-localize at the pole (Fig. 6A). The findings suggested that BdlA moves toward DipA in response to the dispersion signal, resulting in their co-localization at the flagellated pole.

To determine whether BdlA movement toward the flagellated pole is DipA-dependent, we examined BdlA foci formation in biofilms formed by the Δ*dipA* mutant in the absence or presence of nitric oxide. As in wild-type biofilms, BdlA-GFP remained diffusely distributed in untreated Δ*dipA* biofilms, with ~10% of cells exhibiting BdlA foci (Fig. 6B). However, whereas nitric oxide increased BdlA foci formation to ~58% in wild-type biofilms (Fig. 5C and 6C), Δ*dipA* biofilms showed only a modest increase to ~25% following nitric oxide exposure (Fig. 6C). A similar reduction in BdlA foci formation relative to wild-type biofilms was observed in *cheA* mutant biofilms upon nitric oxide exposure (Fig. 6C).

The results indicate that the movement of BdlA toward the flagellated pole in response to the dispersion signal requires DipA (and CheA), with DipA (and CheA-dependent polarity) being necessary for efficient BdlA relocalization post-induction of dispersion.

Given the DipA-dependence of BdlA foci formation, we were surprised to observe that BdlA formed multiple foci per cell (Fig. 5A; Fig. S4). Quantitative analysis showed that most biofilm cells contained a single BdlA focus both before and after nitric oxide treatment (Fig. 6D). However, nitric oxide exposure led to the appearance of additional foci, with the proportion of cells harboring two BdlA foci increasing from ~1% to ~22% and those with three foci increasing from 0 to ~3.5% over the course of 30 min (Fig. 6D). These foci localized not only to the poles but also to mid-cell positions. Representative examples of cells containing two or three BdlA foci are shown in Fig. 6E (see BdlA-GFP). To determine whether these additional foci were linked to DipA, we examined BdlA-GFP and DipA-mCherry co-localization in cells exposed to nitric oxide. In biofilm cells harboring two or three BdlA foci, all BdlA foci co-localized with DipA (Fig. 6E), indicating that the formation of multiple BdlA foci remains DipA-dependent.

### Relocation of BdlA coincides with a reduction in c-di-GMP but an increase in *fliC* expression levels

Dispersion has been associated with an overall decrease in the c-di-GMP level (7, 17, 21). Previous studies have also shown that activated BdlA interacts with DipA and stimulates its PDE activity, thereby lowering c-di-GMP upon induction of dispersion (7, 13, 16). Here, we demonstrated that BdlA forms foci and relocalizes to polar DipA in response to dispersion signals (Fig. 5C), and that active BdlA, together with DipA PDE activity, drives elevated *fliC* expression (Fig. 3D; Fig. S5).

To determine whether BdlA foci formation and its co-localization with DipA are linked to changes in c-di-GMP levels and *fliC* expression upon induction of dispersion, we generated two reporter strain combinations. To simultaneously track BdlA foci and c-di-GMP levels, we used biofilm cells either expressing *bdlA-gfp* or the unstable c-di-GMP reporter PcdrA-mGL(ASV); to monitor c-di-GMP levels alongside *fliC* expression, we used biofilms expressing dual reporters composed of the unstable reporter PcdrA-mGL(ASV) and the *fliC* reporter PfliC*-*mScarlet (ASV). Biofilms formed by the reporter strains were harvested following nitric oxide exposure (or left untreated) and then spotted onto agar pads at 10-min intervals to assess either BdlA cluster formation with c-di-GMP reporter activity or c-di-GMP levels concurrently with *fliC* expression.

**Fig 7:**
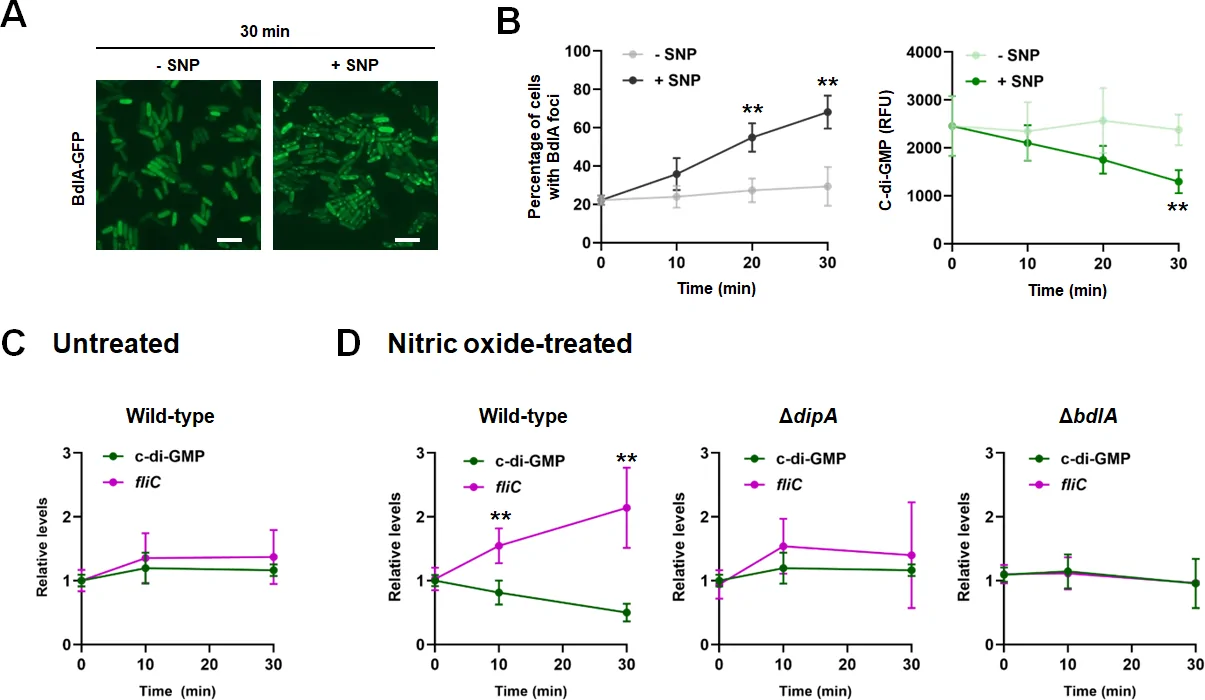
Relocation of BdlA leads to a reduction in c-di-GMP but an increase in *fliC* expression levels. Tube reactor-grown biofilms were harvested and either exposed to SNP or left untreated and then spotted onto agarose pads at 10-min intervals. (**A and B**) Time-dependent changes in BdlA cluster formation and c-di-GMP/cAMP levels in response to the dispersion signal nitric oxide (SNP) were monitored using *P. aeruginosa* PAO1 cells harboring a plasmid expressing *bdlA-gfp* or the unstable c-di-GMP/cAMP reporter plasmid, XZ385, respectively. (**A**) Representative images of BdlA relocation after 30 min of incubation with and without SNP. Bars, 5 µm. (B, left) The percentage of cells exhibiting BdlA-GFP was assessed at each time point. Statistical significance relative to time = 0 was assessed using one-way analysis of variance followed by a Šídák’s multiple comparisons test. **, *P*  <  0.001. (B, right) The c-di-GMP reporter activity at each time point was calculated as background-subtracted fluorescent intensity divided by the amount of bacteria. Statistical significance relative to time = 0 was assessed using two-way analysis of variance. **, *P*  <  0.001. (**C and D**) Time-dependent changes in c-di-GMP and *fliC* expression levels in response to SNP. *P. aeruginosa* PAO1 wild-type, Δ*dipA*, or Δ*bdlA* mutant cells harboring the unstable c-di-GMP/*fliC* reporter plasmid (XZ271) were monitored. (**C**) Untreated wild-type cells served as the control. (**D**) Reporter activities of the wild type and each mutant strain were assessed at indicated time points following SNP treatment. (**B–D**) At least three images per replicate, representing a total of 1,000 cells, were analyzed. All experiments were performed at least in triplicate. Statistical significance relative to time = 0 was assessed using two-way analysis of variance. **, *P*  <  0.001.

Nitric oxide exposure increased BdlA foci to ~55% within 20 min and >70% within 30 min, whereas untreated biofilms showed no change (Fig. 7A and B). Over the same period, nitric oxide triggered a marked threefold decrease in c-di-GMP reporter activity, a response absent in controls (Fig. 7B). A similar response in c-di-GMP reporter activity was noted for biofilms expressing PcdrA-mGL(ASV) and PfliC*-*mScarlet (ASV) in the presence of nitric oxide, whereas untreated biofilms showed no change (Fig. 7C and D). While c-di-GMP reporter activity decreased, *fliC* reporter activity increased markedly over the same time frame in this strain, a response absent in untreated biofilms (Fig. 7C and D).

The findings indicated that BdlA relocalization, the drop in c-di-GMP, and the induction of *fliC* expression are coordinated events triggered by the dispersion signal. To determine whether the nitric oxide–induced decrease in c-di-GMP and the simultaneous increase in *fliC* expression require BdlA, we examined c-di-GMP and *fliC* reporter activity in biofilms formed by the Δ*bdlA* mutant. In this background, nitric oxide failed to alter either c-di-GMP or *fliC* reporter activity (Fig. 7D), indicating that both responses depend on BdlA. Likewise, biofilms formed by the Δ*dipA* mutant showed no significant change in c-di-GMP or *fliC* reporter activity in the presence or absence of nitric oxide (Fig. 7D), demonstrating that DipA is also required for these dispersion-associated signaling outputs.

Taken together, these results demonstrate that BdlA relocalization, the drop in c-di-GMP, and induction of *fliC* form a tightly coordinated cascade that is initiated by BdlA and executed through DipA. Moreover, our results indicate that both BdlA and DipA are essential for linking dispersion-signal sensing to downstream transcriptional and second-messenger responses.

## DISCUSSION

This study examined the motility switch underlying biofilm dispersion by dissecting early regulatory events leading to *fliC* expression and the restoration of flagella-driven motility in dispersed cells. Microscopy revealed a pronounced increase in flagellar gene expression at sites of dispersion (Fig. 2A). Moreover, elevated *fliC* expression during biofilm dispersion (Fig. 1D) was linked to reduced c-di-GMP levels, mediated by BdlA and the PDE DipA (Fig. 3A, 6 and 7).

**Fig 8:**
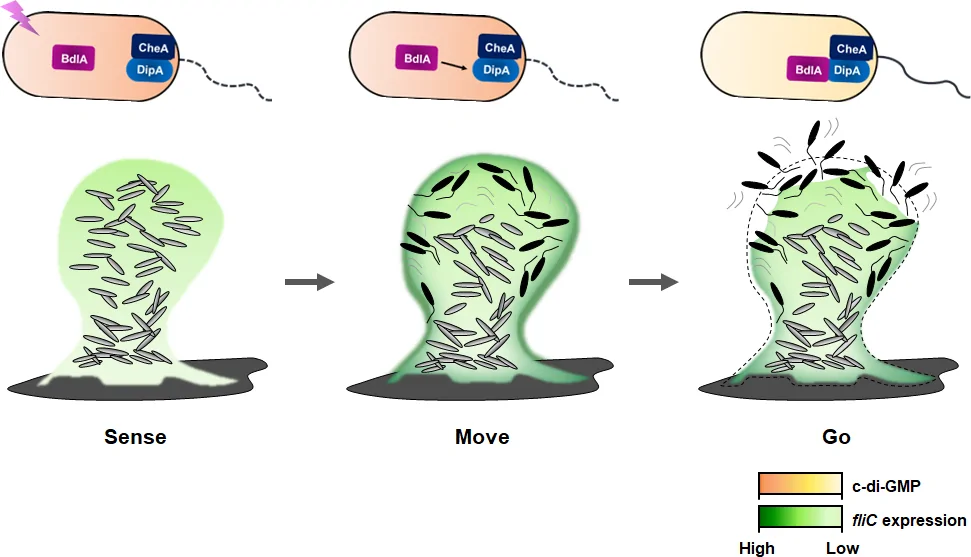
Sense-Move-Go model for *P. aeruginosa* biofilm dispersion. In response to the dispersion signal (Sense), BdlA forms dynamic clusters and relocates to the flagellated pole (Move), where it interacts with and stimulates the PDE activity of DipA. It leads to a decrease in cellular c-di-GMP levels and an upregulation of *fliC* expression, enabling *P. aeruginosa* cells to regain flagella-driven motility. Ultimately, these cells are able to egress from mature biofilms and transition to a free-living lifestyle (Go).

Kulasekara et al. (33) previously demonstrated that polar DipA localization drives the bimodal distribution of c-di-GMP in daughter cells during *P. aeruginosa* division, thereby influencing flagellar motility. Laventie et al. (34) further proposed the Touch-Seed-and-Go model of asymmetric surface division. The model entails flagellated mother cells attach (Touch), divide, and generate two types of daughter cells: non-flagellated daughter cells with high c-di-GMP (Seed) and flagellated daughter cells that maintain polar DipA and low c-di-GMP, detach, and resume swimming (Go). In contrast, non-flagellated daughters lack polar DipA, accumulate c-di-GMP, and recruit the effector FimW to the pole (34). Polar FimW promotes Type IV pili assembly, anchoring cells to the surface (34). This motility-switch mechanism appears restricted to the reversible attachment stage, and its regulation beyond this stage during biofilm development remains unresolved. By examining early regulatory events in the *P. aeruginosa* dispersion response, we found that polar DipA contributes to flagellar motility during dispersion, consistent with the Touch-Seed-and-Go model (33–35). Notably, however, our results reveal deviations from this model, particularly in the spatiotemporal regulation of BdlA and DipA. Our data suggest that BdlA relocation stimulates DipA’s PDE activity, thereby promoting flagellar motility during biofilm dispersion (Fig. 8). In response to the dispersion signal (Sense), BdlA forms dynamic clusters and relocates to DipA at the flagellated pole (Move), leading to reduced c-di-GMP levels and induction of *fliC* expression (Figure-model). Thus, the spatiotemporal coordination of BdlA and DipA drives the transition from a sessile biofilm to a motile, dispersed phenotype (Go). These aspects are captured in our proposed Sense-Move-and-Go model (Fig. 8), which contrasts with the Touch-Seed-and-Go mechanism regulating motility upon surface contact (34, 35).

In both models, DipA’s polar PDE activity drives cell release from surfaces and biofilms during reversible attachment and enables dispersion. While the mechanism of silencing DipA’s PDE activity during other biofilm developmental stages, such as irreversible attachment and biofilm maturation, remains unclear, our study demonstrates that BdlA restores or activates DipA’s PDE activity, thereby enhancing flagellar gene expression upon the induction of dispersion. Notably, *fliC* expression was markedly elevated at the periphery of microcolonies, which are more exposed to dispersion signals (Fig. 2A; Fig. S1). Furthermore, exposure of biofilm cells to the dispersion signals nitric oxide and citrate coincided with BdlA foci formation and polar relocation (Fig. 5 to 7; Fig. S3), a response that was accompanied by decreased c-di-GMP levels (Fig. 7).

Previous study indicated that post-translational, non-processive proteolysis of BdlA is required for the dispersion response of *P. aeruginosa* and is induced in dispersed but not in planktonic cells (15). Similarly, our results demonstrated that BdlA relocates in response to nitric oxide in biofilm cells but not in planktonic cells (Fig. 5). We also showed that relocation and activation of BdlA lead to a shift in c-di-GMP levels and *fliC* expression (Fig. 7), indicating that both relocation and processing of BdlA are required for the dispersion response. In contrast, Δ*bdlA* and Δ*dipA* mutants failed to decrease c-di-GMP or induce *fliC* in response to nitric oxide (Fig. 7), indicating that both BdlA and DipA are essential for coupling dispersion-signal sensing to downstream transcriptional and second-messenger responses. Thus, BdlA relocalization, the subsequent drop in c-di-GMP, and induction of *fliC* form a coordinated signaling cascade that is initiated by BdlA and executed through DipA. Together, the data position BdlA as the upstream regulator that recruits and activates DipA, thereby driving the c-di-GMP decline required for motility gene induction during dispersion (Fig. 8). However, our data revealed that processing-deficient BdlA still forms foci (Fig. S4), indicating that BdlA is likely relocated prior to being processed to become active. Collectively, these findings underscore that *P. aeruginosa* dispersion is a highly regulated process, in which mode of growth-specific and sequential post-translational control of BdlA is essential.

Our research shows that early regulatory events in dispersion coincide with enhanced flagellar gene expression, enabling dispersed cells to regain flagella-driven motility through the coordinated roles of BdlA and DipA. Although the precise mechanisms by which active BdlA stimulates DipA’s PDE activity and how DipA specifically modulates flagellar gene expression in response to dispersion signals remain unresolved, our data clearly demonstrate that BdlA moving to polar DipA in response to dispersion signals reduces c-di-GMP levels, thereby inducing *fliC* expression. In conclusion, we reveal a regulatory mechanism in which BdlA and DipA orchestrate the motility switch during *P. aeruginosa* dispersion, linking signal sensing to cellular processes and phenotypic change.

## MATERIALS AND METHODS

### Bacterial strains, media, and culture conditions

*P. aeruginosa* strain PAO1 and isogenic mutants used in this study are listed in Table 1. In-frame deletion mutants used in this study were generated by allelic exchange using the pEX18-Gm plasmid, as previously described (37). Planktonic cells were grown in Luria-Bertani (LB) medium or Vogel-Bonner minimal medium (VBMM) (38) at 37°C under shaking conditions (220 rpm). Biofilms were grown as indicated below. The following supplements were added if necessary: tetracycline, 60 μg mL^−1^ for *P. aeruginosa* and 15 μg mL^−1^ for *Escherichia coli*; gentamicin, 50 μg mL^−1^ for *P. aeruginosa* and 15 μg mL^−1^ for *E. coli*; carbenicillin, 250 μg mL^−1^ for *P. aeruginosa*; ampicillin, 100 μg mL^−1^ for *E. coli*; and L-arabinose, 0.05%–0.1%.

**TABLE 1:** Strains and plasmids used in this study

Strains or plasmids	Genotypes and/or descriptions^*a*^	Reference or source
Strains		
*P. aeruginosa*		
PAO1	Wild-type PAO1	B.H. Holloway
Δ*fliC*	Δ*fliC* in PAO1	Gift from M. Parsek
Δ*bdlA*	Δ*bdlA* in PAO1, Km^R^	(15)
Δ*rbdA*Δ*dipA*	PAO1; PA0861::IS (*lacZ* and *tet* were removed by Cre-mediated recombination) and Δ*dipA*	This study
*cheA*::IS	PAO1; PA1458::IS*lacZ*, Tet^R^	Lab stock
*E. coli*		
DH5α	F^-^ φ80*lacZ*ΔM15 Δ(*lacZYA-argF*)U169 *recA1 endA1 hsdR17*(r_k_^−^, m_k_^+^) *phoA supE44 thi-1 gyrA96 relA1 tonA*	New England Biolabs
SM10 λ*pir*	*thi-1 thr leu tonA lacY supE recA*::RP4-2-Tc::Mu λ *pir*, OriT of RP4, Km^R^; conjugational donor	(39)
Plasmids		
pEX18-Gm	Gene replacement vector, *oriT*^+^, *sacB*^+^, MCS from pUC18, Gm^R^	(37)
pCre1	Vector bearing the cre recombinase system, Amp^R^	(40)
pUC18-mini-Tn7T-Gm	Site-specific chromosomal insertion plasmid, Amp^R^, Gm^R^	(41)
pRK2013	Helper plasmid for triparental mating; *mob tra* Km^R^	(42)
pTNS2	Helper plasmid containing site-specific recombinase for chromosomal insertion, Amp^R^	(43)
pJN105	Arabinose-inducible gene expression vector; pBRR-1 MCS; *araC*-P_BAD_, Gm^R^	(44)
pMJT-1	*araC*-P_BAD_ cassette of pJN105 cloned into pUCP18, Amp^R^ (Carb^R^)	(45)
pEX18-dipA	pEX18-Gm-based vector for deletion of *dipA*, Gm^R^	This study
pUC18-mini-Tn7T-gfp(ASV)	Site-specific chromosomal insertion plasmid harboring promoterless *gfp*(ASV), Amp^R^, Gm^R^	This study
pUC18-mini-Tn7T-PfliC-gfp(ASV)	Site-specific chromosomal insertion plasmid harboring P*_fliC_- gfp*(ASV), Amp^R^, Gm^R^	This study
pUC18-mini-Tn7T-PpilA-gfp(ASV)	Site-specific chromosomal insertion plasmid harboring P*_pilA_- gfp*(ASV), Amp^R^, Gm^R^	This study
pJN-GFP	pJN105-based GFP fusion plasmid, Gm^R^	This study
pJN-mCherry	pJN105-based mCherry fusion plasmid, Gm^R^	This study
pJN-*dipA*-GFP	pJN-GFP –based expression plasmid for DipA-GFP, Gm^R^	This study
pJN-*dipA*-mCherry	pJN-mCherry –based expression plasmid for DipA-mCherry, Gm^R^	This study
pMJT-*dipA*-mCherry	pMJT –based expression plasmid for DipA-mCherry, Amp^R^ (Carb^R^)	This study
pJN-*bdlA*-GFP	pJN-GFP –based expression plasmid for BdlA-GFP, Gm^R^	This study
pJN-*bdlA (M130I*)-GFP	pJN-GFP –based expression plasmid for BdlA (M130I)-GFP, Gm^R^	This study
CTX-PbdlA-bdlA-M130I	CTX-PbdlA-*bdlA*-V5/6xHis with an M130I mutation in *bdlA*	(15)
pMJT-*rbdA-*V5/6xHis	C-terminal V5/6xHis-tagged *rbdA* cloned into pMJT-1 at NheI/XbaI, Amp^R^ (Carb^R^)	(46)
pMJT-*dipA-*V5/6xHis	C-terminal V5/6xHis-tagged *dipA* cloned into pMJT-1 at NheI/XbaI, Amp^R^ (Carb^R^)	(17)
pMJT-*dipA* (EAL→AAA) V5/6xHis	C-terminal V5/6xHis-tagged *dipA* with E675A and L677A mutations in pMJT-1	(4)
pBBR1MCS5-PlacP1-mScarletI(ASV)-tet2-PcdrA-mGL(ASV)- t0t1-PrpoD-SCFP3A(ASV)	XZ385, Tri-color reporter measuring the promoter activity of lacP1, cdrA, and rpoD, Gm^R^	(47)
pBBR1MCS5-PfliC-mScarlet(ASV)-tet2-PcdrA-mGL(ASV)-t0t1-PrpoD-SCFP3A(ASV)	XZ271, Tri-color reporter measuring the promoter activity of fliC, cdrA, and rpoD, Gm^R^	(47)

^*a*^ Km^R^, kanamycin-resistant; Tet^R^, tetracyclin-resistant; Gm^R^, gentamicin-resistant; Amp^R^, ampicillin-resistant; Carb^R^, carbenicillin-resistant.

### Plasmid construction

All plasmids used in this study are listed in Table 1. Oligonucleotides used are listed in Table S1. To generate the transcriptional *fliC* and *pilA* reporter pUC18-mini-Tn7T-PfliC-gfp(ASV) and pUC18-mini-Tn7T-PpilA-gfp(ASV) and the vector control plasmid pUC18-mini-Tn7T-gfp(ASV), we first amplified the *gfp*(ASV) gene by PCR from the pCdrA::gfp(ASV) plasmid (48) using the primers gfp(ASV)-F and gfp(ASV)-R. The PCR product was digested with XhoI and KpnI and inserted into the corresponding sites of pUC18-mini-Tn7T-Gm to generate pUC18-mini-Tn7T-gfp(ASV). To create pUC18-mini-Tn7T-PfliC-gfp(ASV) and pUC18-mini-Tn7T-PpilA-gfp(ASV), the upstream regions of PA1092 (*fliC*) and PA4525 (*pilA*) were amplified by PCR from the chromosome DNA of *P. aeruginosa* PAO1 using primer pairs of PfliC-F/PfliC-R and PpilA-F/PpilA-R, respectively. Two PCR products were cloned into BamHI and XhoI sites of the pUC18-mini-Tn7T-gfp(ASV), resulting in pUC18-mini-Tn7T-PfliC-gfp(ASV) and pUC18-mini-Tn7T-PpilA-gfp(ASV), respectively.

To generate a GFP-tagged DipA construct, the *gfp* gene was amplified by PCR with the primers pJN-GFP-F and pJN-GFP-R. The PCR product was cloned into SacI site of pJN105 to generate pJN-GFP. The *dipA* gene was amplified from the chromosome DNA of *P. aeruginosa* PAO1 by PCR using the primers DipA-F and DipA-GFP-R. This PCR product and a linearized product of pJN-GFP through digestion with NheI and PstI were combined by using the NEBuilder HiFi DNA Assembly Master Mix (New England Biolabs), resulting in the plasmid pJN-*dipA*-GFP expressing the DipA-GFP fusion protein. To generate mCherry-tagged DipA constructs, the *mcherry* gene was amplified by PCR with the primers pJN-mCherry-F and pJN-mCherry-R. The PCR product was cloned into the SacI site of pJN105 to generate pJN-mCherry. The *dipA* gene was amplified from the chromosome DNA of *P. aeruginosa* PAO1 by PCR using the primers DipA-F and DipA-mCherry-R1. This PCR product and a linearized product of pJN-mCherry through digestion with NheI were combined by using the NEBuilder HiFi DNA Assembly Master Mix (New England Biolabs), resulting in the plasmid pJN-*dipA*-mCherry expressing the DipA-mCherry fusion protein. To create pMJT-*dipA*-mCherry, the *dipA-mcherry* portion from pJN-*dipA*-mCherry was amplified by PCR with the primers DipA-F and DipA-mCherry-R2. The PCR product and a linearized pMJT-1 through digestion with NheI and SacI were combined by using the NEBuilder HiFi DNA Assembly Master Mix (New England Biolabs).

To generate GFP-tagged BdlA and BdlA (M130I) constructs, the *bdlA* and *bdlA (M130I*) genes were amplified from the chromosome DNA of *P. aeruginosa* PAO1 and the plasmid CTX-PbdlA-bdlA-M130I, respectively, by PCR using the primer pair BdlA-GFP-F/-R. Each PCR product and a linearized pJN-GFP through digestion with NheI were combined by using the NEBuilder HiFi DNA Assembly Master Mix (New England Biolabs), resulting in the plasmids pJN-bdlA-GFP and pJN-bdlA (M130I)-GFP.

### Biofilm growth

Biofilms were grown in fivefold-diluted VBMM medium using a continuous-flow tube reactor system with a size of 13 for biofilm biomass accumulation for 3 days and 14 Masterflex silicone tubing for dispersion assays for 5 days (Cole Parmer Inc.) at flow rates of 0.1 and 0.2 mL min^−1^, respectively (5, 12, 49). To visualize the biofilm architecture prior to and after induction of dispersion, biofilms were grown in flow cells (glass surface, BioSurface Technologies) at a flow rate of 0.2 mL min^−1^, using fivefold-diluted VBMM medium (15, 16). For biofilm cultivation in a 24-well plate, biofilms were grown in 5-fold-diluted LB medium, as previously described (49, 50). The plate was incubated at 37°C and 220 rpm at a 45° angle, with the growth medium exchanged every 12 h. Where applicable, the following supplements were added to the growth medium: carbenicillin, 10 μg mL^−1^; gentamicin, 2 μg mL^−1^; and L-arabinose, 0.05%.

### Biofilm dispersion

Biofilm dispersion assays were performed as previously described (12, 15, 16, 49). Briefly, dispersion of 5-day-old biofilms, grown in continuous flow tube reactors, was induced by adding 0.5 mM sodium nitroprusside (SNP), 20 mM sodium citrate, or 3% L-arabinose to the growth medium. Then, dispersed cells were collected from the tube reactor effluents into 96-well plates at 1-min intervals for up to 35 min. The absorbance of the biofilm effluents was measured at 600 nm using spectrophotometry. An increase in the turbidity of the effluent indicated dispersion events. To visualize induced dispersion, dispersion of flow-cell-grown 4-day-old biofilms was induced in a similar manner and monitored by confocal laser scanning microscopy using a Leica TCS SP5 confocal microscope (Leica Microsystems, Inc.). Confocal images prior to and following induction of dispersion were analyzed using COMSTAT as previously described (15, 16) to determine *fliC*/*pilA* expression and biomass changes. For spatial distribution analysis of *fliC* expression, line-scan analysis was performed using ImageJ (51). The relative GFP intensity was measured from the center to the periphery of the microcolonies. Regression lines were fitted to the pooled data points from three independent microcolonies. Experiments were performed in triplicate using biological replicates. For quantitative image analysis, at least eight images per replicate were examined.

### RNA isolation and qRT-PCR

Total RNA was isolated from tube reactor-grown 5-day-old biofilm cells and SNP-exposed dispersed cells using the E.Z.N.A. Total RNA kit (Omega Bio-Tek) according to the manufacturer’s instructions. Genomic DNA removal and cDNA synthesis were performed as previously described (50). qRT-PCR was carried out using the BioRad CFX Connect real-time PCR detection system (Bio-Rad) and SsoAdvanced SYBR Green Supermix (Bio-Rad) with oligonucleotides listed in Table S1. The *cysD* gene was used as a reference to normalize the transcript level.

### Analysis of flagella and pili gene expression

Relative flagella and pili expression levels of biofilms grown under flowing conditions in tube reactors and dispersed cells were determined using translational fusions of *fliC* (flagella) and *pilA* (pili) to the unstable GFP, GFP(ASV), reporters. Biofilms were harvested, resuspended in phosphate-buffered saline (PBS), and the resulting suspension was homogenized to ensure disaggregation. Dispersed cells were obtained using SNP as described above, washed with PBS, and homogenized. The absorbance (600 nm) and fluorescence (GFP, excitation 485 nm/emission 535 nm) of biofilm and dispersed cells were measured in a 96-well black clear-bottom microtiter plate (Greiner Bio-One) using a SpectraMax i3x plate reader (Molecular Devices). Quantifications were performed in triplicate using biological replicates, and the fluorescence unit from GFP was normalized to absorbance.

### Visualization of fluorescent fusion proteins and relative levels of c-di-GMP and *fliC* in live cells

Two approaches for somewhat immobilizing cells to visualize fusion proteins (DipA and BdlA) and reporter activity (fliC and cdrA/c-di-GMP) were used. First, planktonic, dispersed, and biofilm cells were spotted on an agarose pad (1% [wt/vol] of agarose in fivefold-diluted VBMM). The cells were not OD-adjusted or vortexed. Second, to examine responses to nitric oxide and citrate, planktonic and biofilm cells were harvested and resuspended in PBS to an optical density (OD_600_ of ~1). The cell suspension was split, with half serving as controls and half being subjected to 0.5 mM SNP or 20 mM sodium citrate for the indicated time. Then, 2 μL of the suspension was then spotted on the agarose pad. For both approaches, fluorescent images were acquired using either an Olympus BX60 microscope and 100× UPlanF Olympus objectives or the Leica TCS SP5 confocal microscope (Leica Microsystems, Inc.). The microscopy images were processed using ImageJ (51) and the plugin MicrobeJ (52, 53). To quantify c-di-GMP and/or *fliC* reporter activities, individual bacteria were first identified as rod-shaped particles using MicrobeJ. Then, the background-extracted fluorescence intensities of mGreenLantern (mGL) and mScarlet were measured using ImageJ and normalized to the total cell count. For quantitative analysis, at least 3 images per replicate representing a total of 1,000 cells were examined. For comparative analysis (e.g., time course), all images presented in one figure were obtained under the same conditions and processed using the same settings.

### Statistical analysis

All statistical analyses were performed in GraphPad Prism 9. Unless otherwise noted, all experiments were performed at least in triplicate using biological replicates.

## Data Availability

All data supporting the findings of this study are available within the paper and its Supplemental material. We will make data fully available and without restriction upon request.

## SUPPLEMENTAL MATERIAL

Supplemental table and figures jb.00022-26-s0001.pdf Table S1 and Fig. S1 to S6.

ASM does not own the copyrights to Supplemental Material that may be linked to, or accessed through, an article. The authors have granted ASM a non-exclusive, world-wide license to publish the Supplemental Material files. Please contact the corresponding author directly for reuse.
