# Supplementary material for: Biofilm dispersion by Pseudomonas aeruginosa requires relocation of BdlA to drive a switch in motility by dispersed cells
Source: J Bacteriol. 2026 Aug 12;208(9):e00022-26. doi: 10.1128/jb.00022-26 (PMC13584717; doi:10.1128/jb.00022-26)
Supplement: Supplementary file 1 [file jb.00022-26-s0001.pdf]

Supplemental Material

Biofilm dispersion by *Pseudomonas aeruginosa* requires relocation of BdlA to drive a switch in motility by dispersed cells

Soyoung Park[1,2] and Karin Sauer[1,2#]

[1] Department of Biological Sciences, Binghamton University, Binghamton, New York, USA

[2] Binghamton Biofilm Research Center, Binghamton University, Binghamton, New York, USA

SUPPLEMENTAL TABLES

Supplemental Table S1. Oligonucleotides used in this study

Name	Oligonucleotide sequence (5’–3’)	Use(s)
gfp(ASV)-F	CTATGAGCCTCGAGATGCGTAAAGGAGAAGAAC (XhoI)	Cloning/reporter
gfp(ASV)-R	TTCGCGAGGTACCTTAAACTGATGCAGCGTAGT (KpnI)	
PfliC-F	CTCACTAGTGGATCCGGGTTTTTCTCGAACGAGGCCGGTGGC (BamHI)	
PfliC-R	CCTTTACGCATCTCGAGGGTGATTTCCTCCAAAGGACCTATTTCGG (XhoI)	
PpilA-F	CTCACTAGTGGATCCTGTCGTTCATGGGGAAGGAATCGCAGAAGG (BamHI)	
PpilA-R	CCTTTACGCATCTCGAGGAATCTCTCCGTTGATTATGTATAGGCC (XhoI)	
Del_dipA_Left-F	GGCCAGTGCCAAGCTTCCGCTGTCGCTGTCCTAC (HindIII)	Cloning/deletion
Del_dipA_Left-R	GATAGCGGGATCCGGCGGCATCGGGATGAC (BamHI)	
Del_dipA_Right-F	TGCCGCCGGATCCCGCTATCCCTGCCGCAC (BamHI)	
Del_dipA_Right-R	TTACGAATTCGAGCTCACCGAGCGGTACTGGGTG (SacI)	
pJN-GFP-F	CGCGGTGGAGCTCATGAGTAAAGGAGAAGAAC (SacI)	Cloning/localization
pJN-GFP-R	CGGCGGTGAGCTCCTATTTGTATAGTTCATCCATGC (SacI)	
pJN-mCherry-F	CGCGGTGGAGCTCATGGTGAGCAAGGGCGAG (SacI)	
pJN-mCherry-R	CGGCGGTGAGCTCCTACTTGTACAGCTCGTCCATG (SacI)	
DipA-F	CCCGTTTTTTTGGGCTAGCATGAAAAGTCATCCCGATGCCGCC (NheI)	
DipA-GFP-R	GTGGATCCCCCGGGCTGCAGGTGCAGGGTGCGGCAGGGATAG (PstI)	
DipA-mCherry-R1	GGCTGCAGGAATTCGCTAGCGTGCAGGGTGCGGCAGGGATAG (NheI)	
DipA-mCherry-R2	GACCATGATTACGAATTCGAGCTCCTACTTGTACAGCTCGTCCATG (SacI)	
BdlA-GFP-F	CCCGTTTTTTTGGGCTAGCATGGCGGCCCTGGACCGCTCCATG (NheI)	
BdlA-GFP-R	GGCTGCAGGAATTCGCTAGCGAGATCGGCGTTGAGGGTGCGC (NheI)	
fleQ_F	TGGCGCGAAACCAAACTCTTGC	qRT-PCR
fleQ_R	CTTCAGCAGCTCTACCGCACC	
flhF_F	GGACCGTCAATTGTTCGCCGAG	
flhF_R	GGCGATGGAACCCAGTTGTACC	
fliG_F	TTACGAACACCCGCAGATCCAGG	
fliG_R	GATCAGGTTGAGTTCCTTCAGCGCC	
flgB_F	CACCACCAACGAGCGGCATATC	
flgB_R	CTGTTGAGGAAGGTGAAGGACGCC	
flgF_F	GAAGCAGATGGAGAAGGGTACCG	
flgF_R	TGCAGCTCGAATTGACGGGACAG	
flgK_F	GCCAGATCGACCAGCTCAACTC	
flgK_R	CTTGTCCAGTTGGTCGTAGAGGG	

cheA_F	GACGATATGGACCTGCTCAATGCC	
cheA_R	GCAGCACCACATCCATCAGTTCG	
motC_F	TCAGCCTGGTCGGGATCATC	
motC_R	GAAGAACACCCAGCCGAGCATG	
fliC_F	CCTACGAGACCATTGACATCAGCC	
fliC_R	CGATGGCGATGTTCTTCACCTGAC	
cysD_F	CTGGACATCTGGCAATACAT	
cysD_R	TCTCTTCGTCAGAGAGATGC	

* Engineered restriction sites were underlined with the corresponding restriction enzyme shown in parentheses.

SUPPLEMENTAL FIGURES

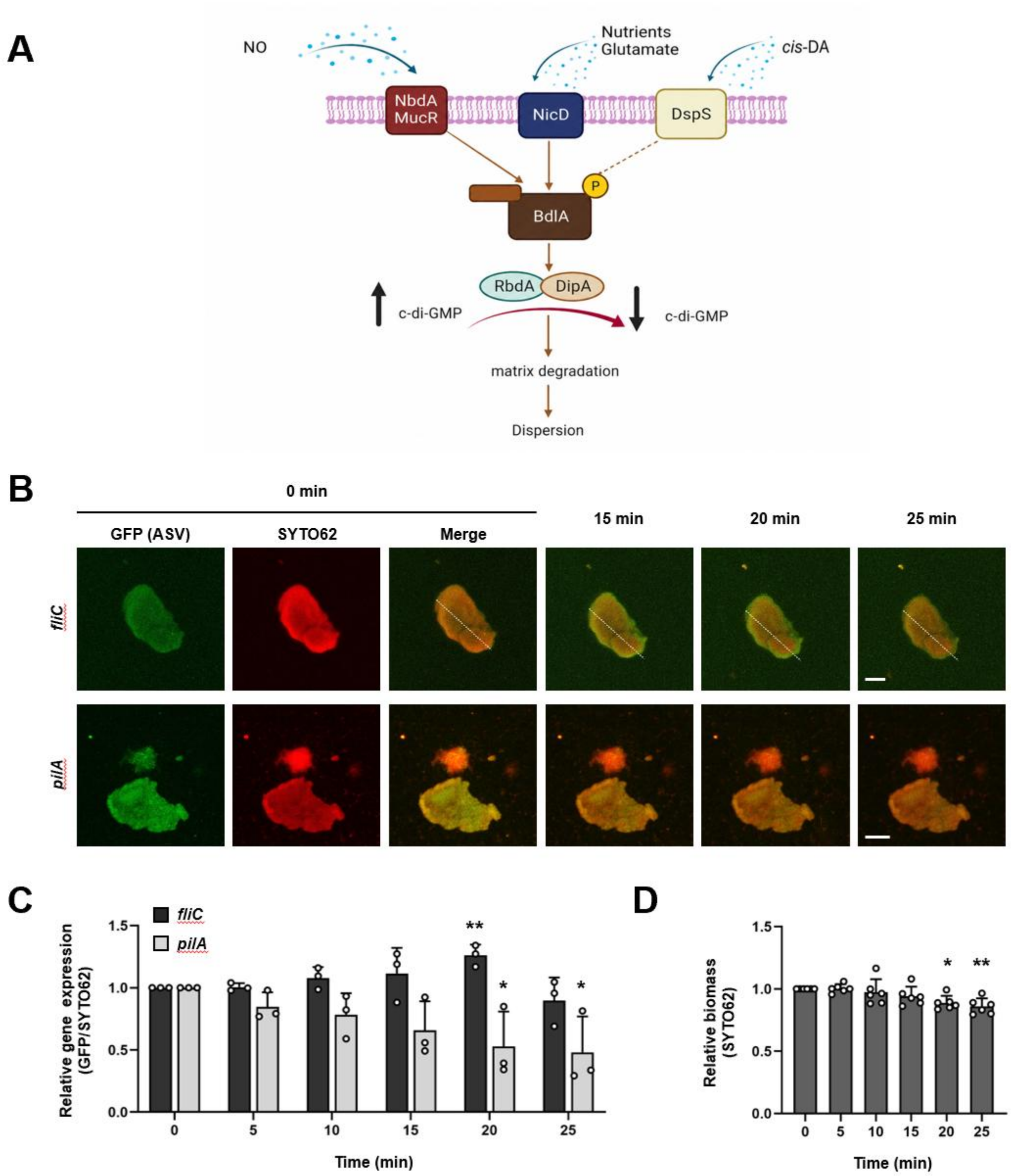

Supplemental Figure S1. Model of dispersion pathways, and expression patterns of *fliC* and *pilA* within microcolonies upon induction of dispersion. (A) Model of sensory pathways governing biofilm dispersion. Nitric-oxide is detected by NbdA and MucR, nutrient signals (glutamate, succinate) are sensed by NicD, whereas cis-2-decenoic acid (cis-DA) is sensed by DspS (1-3). Across these dispersion signals, signal sensing triggers non-processive proteolytic activation of BdlA (4). ?, unknown signal relay to BdlA. Activated BdlA recruits the phosphodiesterases DipA and RbdA, shifting the system from high c-di-GMP/low PDE activity to low c-di-GMP/high PDE activity, thereby promoting motility and matrix degradation, which together drive biofilm dispersal. (B-D) Biofilms formed by wild-type PAO1 *fliC* and *pilA* reporter strains were grown for 4 days in 5-fold-diluted VBMM using flow cells under constant flow conditions and stained with SYTO 62 prior to microscopy for biomass. Subsequently, dispersion

was induced by adding 20 mM Sodium citrate, and the biofilms were monitored at 5-min intervals to assess changes in the *fliC* and *pilA* expression. (B) Representative confocal images are shown. The same length of dashed line was present to show a change in biomass. Bars, 50 μm. Time-dependent (C) *fliC* and *pilA* expression and (D) biofilm biomass changes were determined by COMSTAT analysis. Statistical significance relative to time=0 was assessed using an unpaired, two-tailed *t*-test. *, $P < 0.05$.

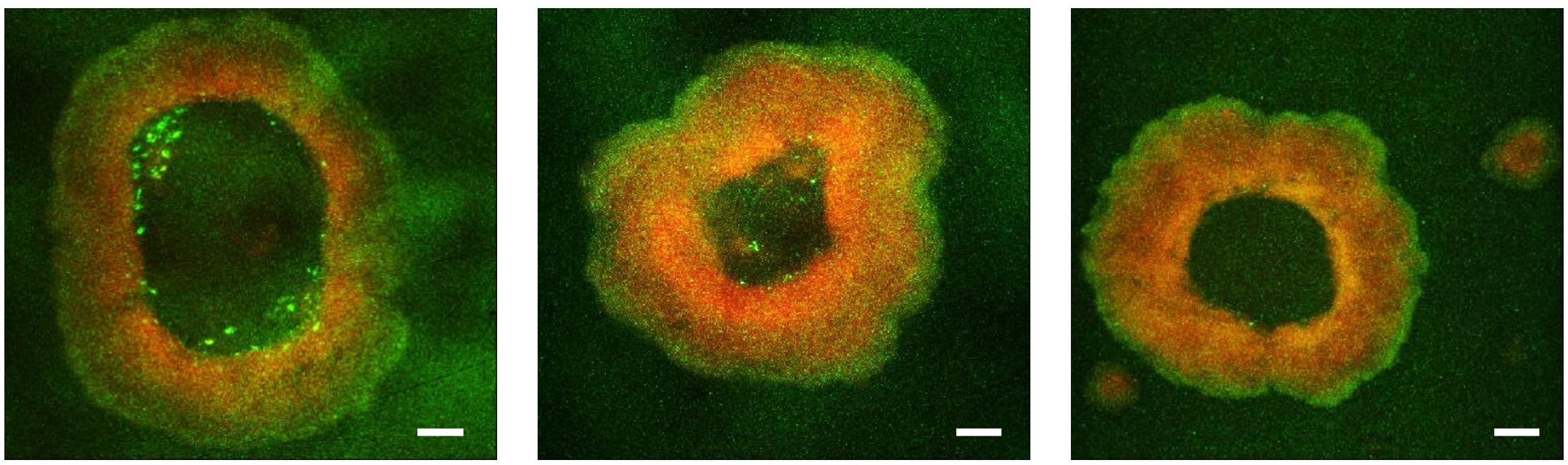

Supplemental Figure S2. Expression patterns of *fliC* within microcolonies during native dispersion. Biofilms formed by *P. aeruginosa* PAO1 *fliC* reporter strain were grown in 5-fold-diluted LB medium for 4 days in 24-well plates. Biofilms were counter-stained with SYTO 62 for biomass and visualized by confocal microscopy. Representative merged images are shown. Bars, 50 μm.

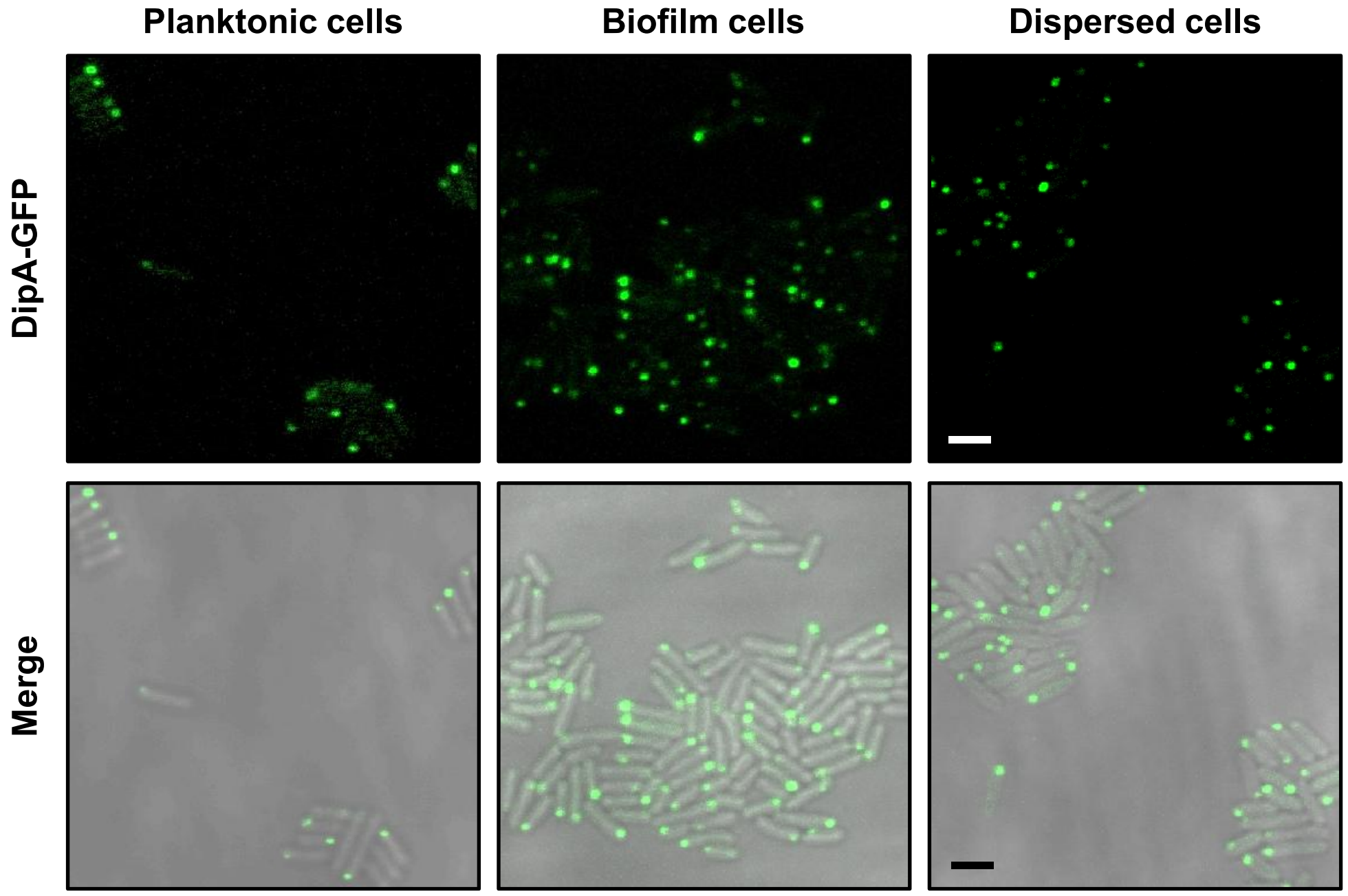

Supplemental Figure S3. Polar localization of DipA in planktonic, biofilm, and dispersed cells. Planktonic cells, biofilm cells grown in a tube reactor, and nitric oxide-induced dispersed cells were placed on an agarose pad to observe DipA distributions. GFP-fused DipA (DipA-GFP) was expressed in wild-type *P. aeruginosa* PAO1, and its subcellular localization was examined using fluorescence microscopy. Representative images are shown. Bars, 2 μm.

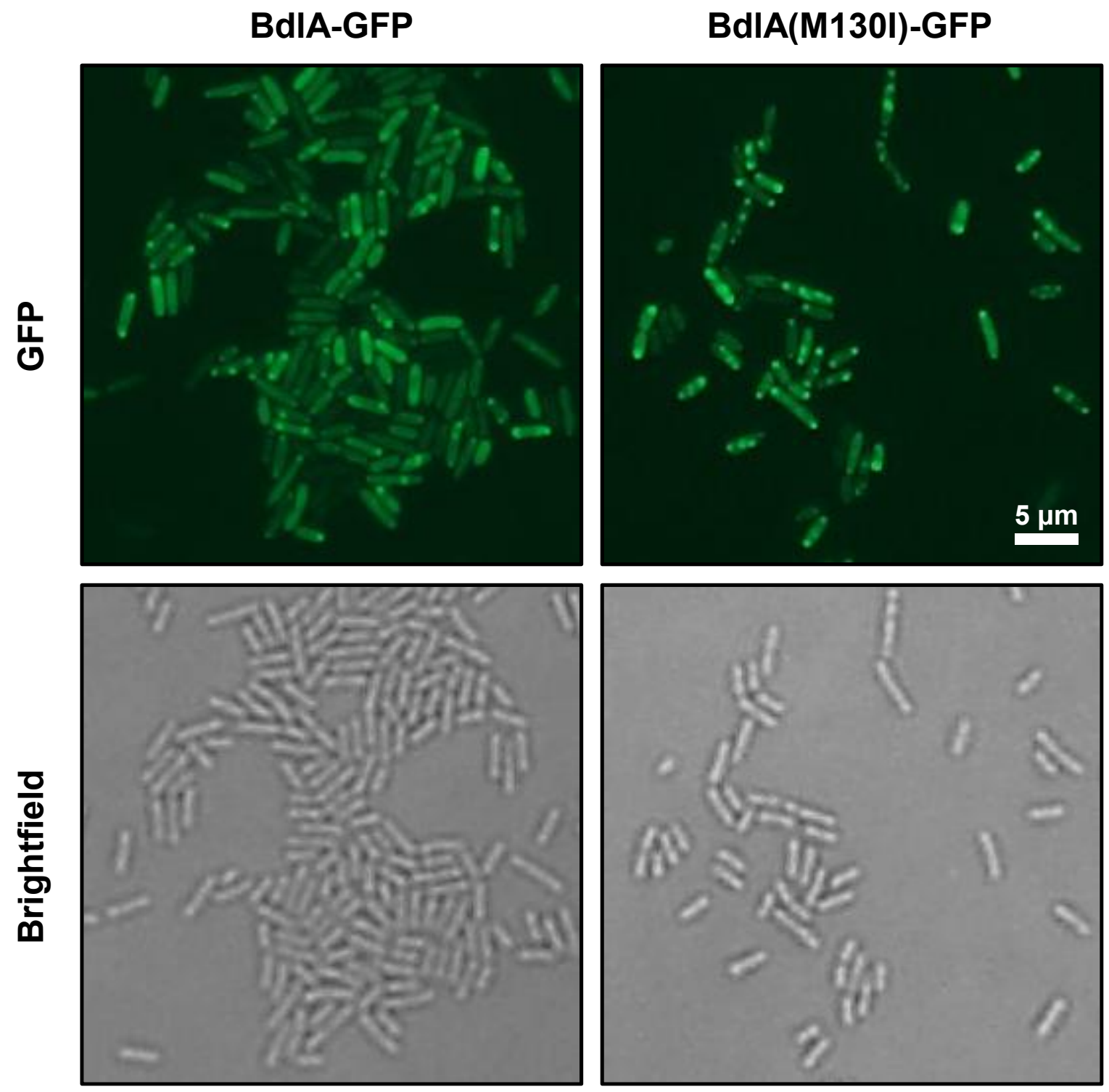

Supplemental Figure S4. Relocation of BdlA and BdlA (M130I) in response to the dispersion signal. GFP-fused BdlA and the BdlA_M130I variant, which is defective in processing and activation, were expressed in wild-type *P. aeruginosa* PAO1, and their localizations were monitored using fluorescence microscopy. Biofilms grown in a tube reactor were harvested and exposed to nitric oxide for 20 min. Representative images are shown. Bar, 5 µm.

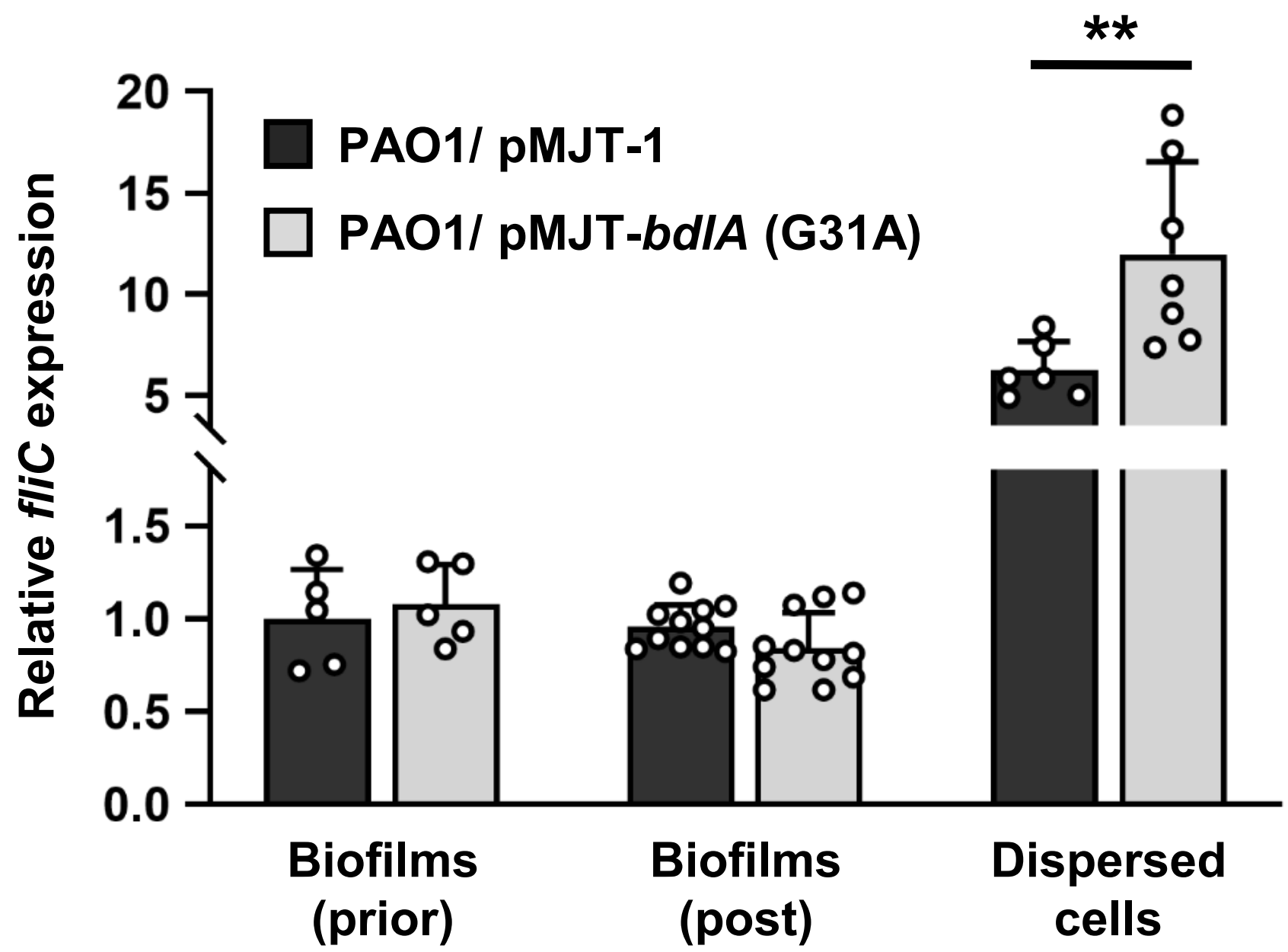

Supplemental Figure S5. Effect of active BdlA on *fliC* expression during biofilm dispersion. Relative *fliC* expression levels were assessed using *fliC* reporter strains harboring either an empty vector or a vector expressing the active form of *bdlA*, *bdlA*_G31A. Biofilm cells were harvested both prior to and after the addition of arabinose, and biofilm effluents containing dispersed cells were collected following arabinose supplementation. Quantifications were performed in triplicate using biological replicates, normalizing GFP fluorescence to absorbance. Statistical significance was assessed using one-way ANOVA: **, $P < 0.001$.

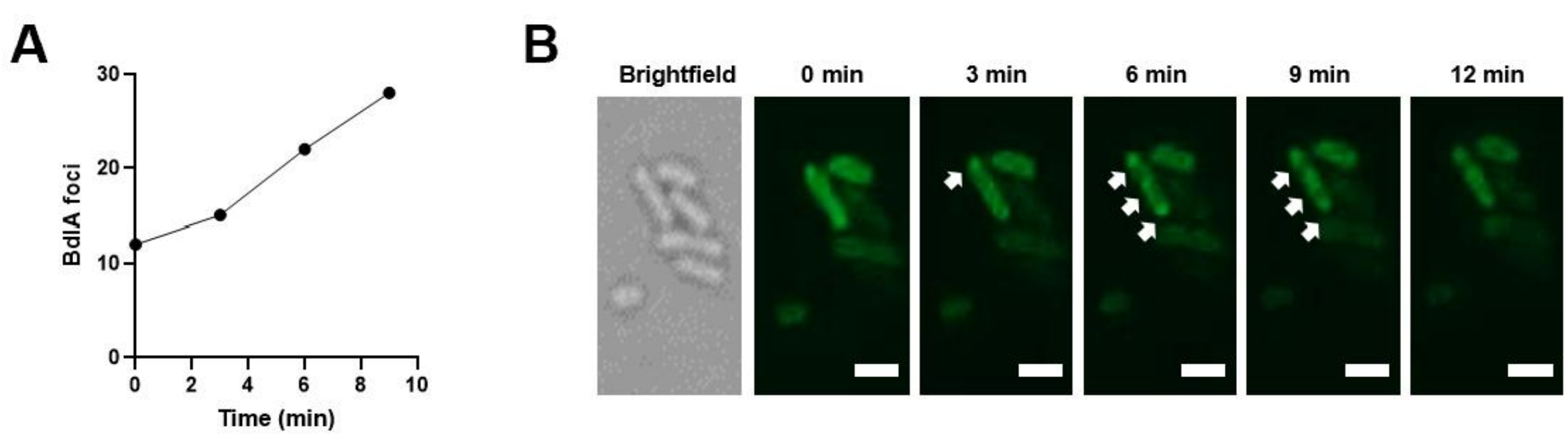

Supplemental Figure S6. Time course of BdlA foci formation. (A) Quantitative analysis of BdlA foci post exposure to nitric oxide, as determined using ImageJ, using microscopic images shown in Figure 5D. (B) Representative images showing time course of multiple BdlA foci per cell post exposure to nitric oxide. White arrows indicate BdlA foci. Bars, 2 µm.

REFERENCES

1. **Kalia M, Amari D, Davies DG, Sauer K.** 2023. *cis*-DA-dependent dispersion by *Pseudomonas aeruginosa* biofilm and identification of *cis*-DA-sensory protein DspS. mBio **0:**e02570-02523.
2. **Basu Roy A, Sauer K.** 2014. Diguanylate cyclase NicD-based signalling mechanism of nutrient-induced dispersion by *Pseudomonas aeruginosa*. Molecular Microbiology **94:**771-793.
3. **Li Y, Heine S, Entian M, Sauer K, Frankenberg-Dinkel N.** 2013. NO-induced biofilm dispersion in *Pseudomonas aeruginosa* is mediated by a MHYT-domain coupled phosphodiesterase. Journal of Bacteriology **195:**3531-3542.
4. **Petrova OE, Sauer K.** 2012. Dispersion by *Pseudomonas aeruginosa* requires an unusual posttranslational modification of BdlA. Proceedings of the National Academy of Sciences **109** 16690-16695.
